# Effects of novel vaccines on weight loss in diet-induced-obese (DIO) mice

**DOI:** 10.1186/2049-1891-3-21

**Published:** 2012-07-09

**Authors:** Keith N Haffer

**Affiliations:** 1 Braasch Biotech LLC, 421 Rose Avenue, Garretson, SD, 570303-0430, USA

**Keywords:** Obesity Treatment, Vaccines, Weight loss, Weight management

## Abstract

The purpose of the study was to test the therapeutic effects of novel vaccines for reducing weight gain and increasing weight loss in diet induced obesity (DIO) model. Male C57BL/6 J mice, fed a 60% Kcal fat diet for 8 weeks prior to the start of the study, were vaccinated via the intraperitoneal route with two formulations (JH17 & JH18) of chimeric-somatostatin vaccines at 1 and 22 days of the study. Control mice were injected with PBS. All mice continued to be feed the 60% Kcal fat diet for the 6 week study. Body weights were measured two times a week and food intake was measured weekly. At week 6, mice were euthanized and a terminal bleed was made and antibody levels to somatostatin and levels of insulin-like growth factor 1 (IGF-1) were determined. Vaccination with both vaccine formulations induced a statistically significant body weight change over the study period, as compared with PBS controls. Percentage of baseline body weight was also significantly affected by vaccination during the study period. Vaccinates finished the study at 104% and 107% of baseline weight, JH17 & JH18 respectively, while untreated controls reached 115% of baseline weight. Food intake per mouse was similar in all mouse groups during the entire study. Control mice did not demonstrate any antibody titers to somatostatin, while all vaccinated mice had measurable antibody responses (> 1:500,000 titer). IGF-1 levels were not statistically significant among the groups, but were elevated in the JH18 vaccinates (mean 440.4 ng/mL) when compared with PBS controls (mean 365.6 ng/mL). Vaccination with either JH17 or JH18 chimeric –somatostatin vaccines produced a statistically significant weight loss as compared with PBS controls (*P* < 0.0001), even though the DIO mice with continually fed a 60% Kcal fat diet. The weight loss/lower weight gain observations were even more significant, as all mice consumed similar amounts of food for the entire study. The presence of high levels of anti-somatostatin antibodies at 6 weeks was correlative with the weight observations and confirmed the success of vaccination.

## Introduction

The prevalence of obesity has risen dramatically in the past decade and is a very serious health problem in the United States, United Kingdom and elsewhere in the world. Obesity is a risk factor for high blood pressure, heart disease, stroke, gall bladder disease, breast cancer, prostate cancer, colon cancer, and type 2 diabetes [1,2]. As a result, extensive biopharmaceutical research is focused upon identifying compounds that can promote weight loss without adversely affecting other aspects of physiology.

One method for pharmacological treatment of obesity is to focus on energy metabolism: appetite and energy intake are both reduced while energy expenditure is maximized. Target candidates for this treatment are growth hormone (GH) and insulin-like growth factor 1 (IGF-1). The latter is secreted by the liver by stimulation of GH and has specific metabolic effects [3]. Treatment with exogenous GH has been reported to have positive effects on obesity in multiple animal models of obesity as well as in human clinical studies [4]. In rat models, the effects of GH treatment accelerated loss of body fat, reduced hypertension and improved cardiovascular function [5,6].

Somatostatin is a 14 amino acid, peptide hormone produced in the hypothalamus as well as certain portions of the digestive system. Somatostatin is known to inhibit the release of GH from the anterior pituitary [7]. Immunization of animals to somatostatin has been recognized as a means of removing somatostatin’s normal inhibitory effects and increasing levels of both GH and IGF-1 [8,9]. Importantly, these somatostatin-based immunization procedures avoid the direct use of anabolic hormones, such as GH or IGF-1[10].

The purpose of the present study was to determine if immunization with somatostatin vaccines in a polygenic, diet-induced-obesity (DIO) mouse model was effective in reducing weight gain and increasing weight loss. The C57BL/6 J (BL) strain is the most commonly used reference strain for diabetes research [11]. The Jackson Laboratory (Harbor, ME) has extensively characterized this mouse strain as to typical weights, plasma glucose levels and percent body fat when fed 10%, 45%, or 60% fat diets [12].

The male C57BL/6 J (BL) mice were continuously fed a 60% Kcal fat diet to test the efficacy of the vaccines without any other contributing factors. In this way, any change in body composition or metabolism could unequivocally be associated with immune response control of somatostatin. We validated the effectiveness of the vaccines by terminal analysis of mouse serum for the presence of antibodies to somatostatin and IGF-1 levels.

## Methods and procedures

Unless stated otherwise all chemicals used in this study were obtained from Sigma Aldrich (St. Louis, MO). All ELISA reagents were obtained from KPL (Gaithersburg, MD). The DIO mouse studies were conducted at The Jackson Laboratory West (Sacramento, CA).

### Animals

Male C57BL 6 J 60% DIO mice were received from The Jackson Laboratory (Bar Harbor, ME) at 10 weeks of age. All mice were acclimated for 2 weeks and then assigned to treatment groups based on body weights. The mice were ear notched for identification using a standard mouse ID format. Mice were housed at a density of 2–4 per cage in polycarbonate cages which were both individually and positively ventilated. The study was conducted according to an IACUC approved protocol and in compliance with the *Guide for the Care and use of Laboratory Animals* (National Research Council, 1996). Filtered tap water acidified to a pH of 2.8 to 3.2 was provided *ad libitum*. High fat diet (D12492) was obtained from Research Diets, Inc (New Brunswick, NJ) and was fed *ad libitum.* Body weights were measured two times per week for 6 weeks. Food intake was measured weekly. Cage side observations were made daily. Mice were euthanized by CO_2_ inhalation at the end of the study. Terminal blood was collected by cardiac puncture and collected into EDTA tubes. Blood was processed to plasma and stored at −80 °C until sent for analysis.

### Vaccines and vaccinations

Vaccines were prepared from purified *E. coli* expressed inclusion body containing chimeric-somatostatin protein, according to “Mendelsohn et al. [13].” Vaccines were formulated with either JH17 or JH18 adjuvants and contained 1 mg protein/mL [14]. The chimeric-somatostatin vaccines will be referred to in the text as simply JH17 and JH18. Vaccines were administered by intraperitoneal injection at day 1 and day 22 of the study. Control mice were vaccinated in a similar manner with PBS. These vaccines had previously been tested and approved for safety and efficacy in a non-obese mouse model.

### IGF-1 measurements

Individual plasma samples were measured with a commercially available mouse/rat IGF-1 sandwich immunoassay ELISA Kit (DSL-10-29200, Diagnostic Systems Laboratories, Inc., Webster, TX). The ELISA is specific for IGF-1 and can detect levels as low as 2 ng/mL. A set of mouse/rat IGF-1 standards were used to generate a standard curve and plasma samples results were fitted onto the standard curve to determine IGF-1 ng/mL. The precision of the assay was reported as a mean coefficient of variation percentage (CV %) and intra-assay CV% was 6.5 while the inter-assay CV% was 5.4.

### Insulin assays

Individual plasma samples were measured with a commercially available Mouse Insulin sandwich immunoassay ELISA Kit (ALPCO 80-INSMS-E01, “E10;” ALPCO Diagnostics, Salem, NH). The ELISA is specific for mouse insulin and the sensitivity of the assay is 0.06 ng/mL. A set of mouse insulin standards were used to generate a standard curve and plasma samples results were fitted onto the standard curve to determine insulin ng/mL. The reported assay precisions were 3.7% (intra-assay) and 3.2% (inter-assay).

### Somatostatin antibody responses

Plasma IgG antibodies, specific for chimeric–somatostatin were determined by an indirect sandwich ELISA. Briefly, Maxisorp plates (Nalgene Nunc, Rochester, NY) were coated with refolded chimeric-somatostatin protein in 0.01 mol/L borate buffer. A 100 μL aliquot of the protein containing 40.5 ng was coated into each well of the plate and allowed to incubate at 2 to 8 °C for 18 hours. After incubation, the plates were emptied and washed once with phosphate buffered saline (pH 7.2) containing 0.05% Tween 20 (PBS-T). All wells were then blocked with 100μL of SIGMA Block and incubated at 37 °C for 1 hour. Plates were then emptied and washed three times with PBS-T. Plasma was diluted from 1:200 to 1:1,600 in Sigma Block. Each dilution of Plasma was added to triplicate wells and incubated for 1 hour at 37 °C. A pool of negative mouse plasma, also at 1:200 to 1:1,600 dilutions, was included on each plate. Plates were then emptied and washed three times with PBS-T. Goat anti-mouse IgG-HRP was diluted to 1:2,000 dilution in PBS-T and 100 μL was added to each well. Plates were incubated for 1 hour at 37 °C. Plates were then emptied and washed three times with PBS-T. Sureblue ™ HRP substrate was added to each well in 100 μL volumes. Reactions were allowed to run for 10 minutes and then stopped with 100 μL of TMB stop solution. Plates were air blanked then read at 450 nm on an ELx800 Reader controlled by KC Junior software (Bio-Tek, Winooski, VT). Linear regression curves for each plasma sample were determined by Microsoft Excel software (Redmond, WA). Endpoint titers were assigned a 0.2 OD and were interpolated from each regression curve for each mouse. The precision of the somatostatin antibody assay was 3.8% and 5.3%, intra-assay and inter-assay CV%, respectively.

### Data analysis

The results are presented as mean ± SEM Comparisons between groups for: body weight means; percent final body weight vs. baseline weight; percent of baseline body weight; and, IGF-1 were conducted using an unpaired two-tailed *t*-test or one-tailed Mann –Whitney (Prism Software; GraphPad San Diego, CA). A *P* value of <0.05 was considered significant. Regression line slope analysis of cumulative food intake was also conducted with Prism Software.

## Results

### Effect of somatostatin vaccines on body weight parameters

**Figure 1:**
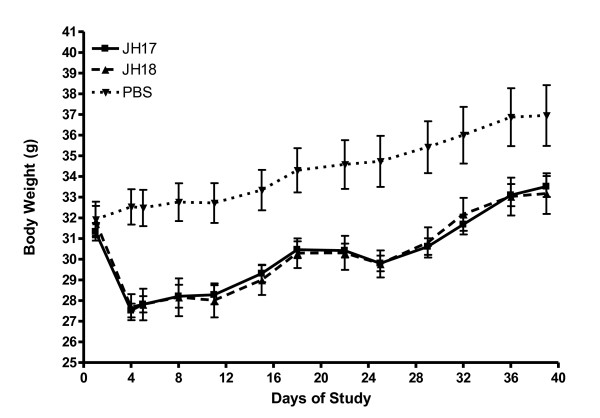
Determination of the effects of vaccination on mean body weight.

Data in Figure 1 demonstrates the effects of vaccination on the mean body weights by treatment group. The original vaccination regime was set at 0.5 mL administered by intraperitoneal injection on days 1 and 22. The initial loss of body weight was first observed at 4 days post- 1^st^ vaccination and was 12.2% and 13.1% for JH 17 and JH18, respectively. Due to the large weight loss, and the concern for the mouse health, the study directors decided to reduce the second vaccination by 1/5^th^ and administer only 0.1 mL on day 22. This second vaccination resulted in an initial weight loss of 2.1% and 1.8% during the 3 days post- 2^nd^ vaccination, JH 17 and JH18 respectively. Statistical determinations of body weight means between JH17 and PBS controls was highly statistically significant by the unpaired two-tailed *t*-test (*P* < 0.0001). The statistical determinations of JH18 versus the PBS controls demonstrated the same statistical significance (*P* < 0.0001). By study date 32, 10 days post-2^nd^ vaccination, both the JH17 and JH18 –vaccinated mice returned to their day 1 baseline body weights, while the PBS controls had gained 4.1 g above their baseline weights. At the end of the study (day 39), there was a weight differential of 3.43 and 3.78 g between the PBS controls and JH17 and JH18 vaccinates, respectively.

**Figure 2:**
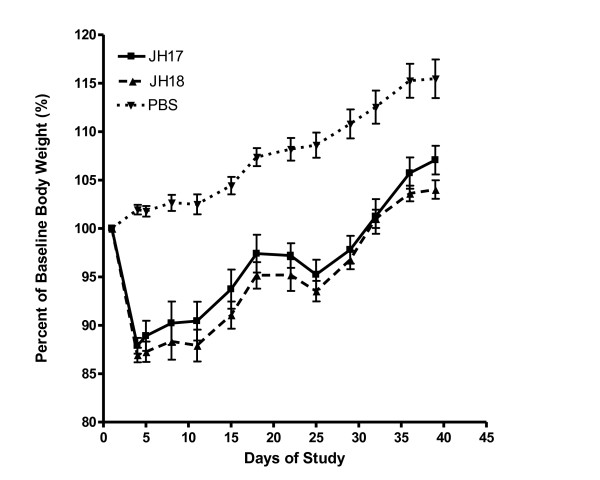
Determination of the effects of vaccination on percent of baseline body weight.

Data in Figure 2 demonstrates the effects of vaccination on the percent of baseline weights by treatment group. This figure depicts baseline weights as 100% and all other weight determinations are compared with this baseline value. Similarly, highly statistical differences were observed with both vaccine groups versus the PBS controls (*P* < 0.0001). At day 39, the mean percent baseline weights were 107.1%, 104.0% and 115.5% for JH17 vaccinates, JH18 vaccinates and PBS controls, respectively. The differences between JH17 and JH18 vaccinates were not statistically significant, although JH18 produced a noticeably lower percent of baseline weight at all post-vaccination days of the study.

### Effect of somatostatin vaccines on food intake

**Figure 3:**
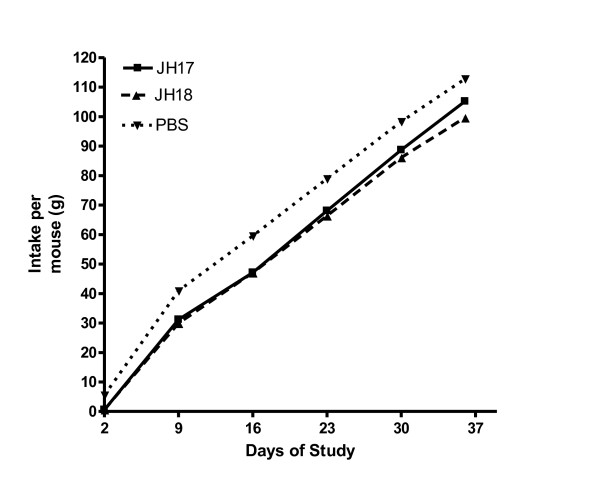
Cumulative food intake of treatment groups.

Data in Figure 3 depicts the cumulative food intake of treatment groups during the study. Values are for each 10 mouse treatment group. Although there was a significant drop in cumulative food consumption for the first 2 days of the study in JH17 and JH18 vaccinates, associated with post-adjuvant injection stress, weekly food consumption through the end of the study was consistent with the PBS control group. Adjuvant controls were not included in this study as no negative effect had previously been observed. At study’s end, the cumulative food intake was 105.2 g for JH17, 99.6 g for JH18 and 113.8 g for PBS controls. Based upon the unpaired t-tests and regression slope analyses, there were not statistically significant differences between either the JH17 or JH 18 mice and the PBS control mice.

### Antibody responses to chimeric-somatostatin vaccine antigen

**Figure 4:**
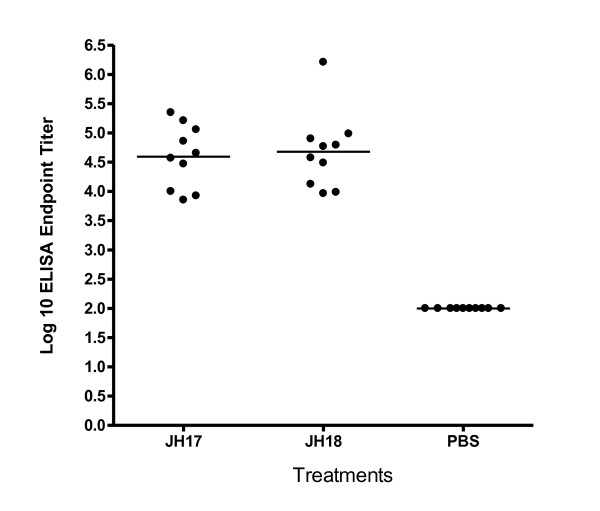
Antibody titers to chimeric-somatostatin in plasma.

Data in Figure 4 demonstrates the immune response after two vaccinations with chimeric-somatostatin protein by ELISA endpoint titers. The PBS controls had no demonstrable titers to somatostatin at the 1:200 initial dilution and were assigned a baseline value of 2 log_10_ (< 1:200 dilution). JH 17 vaccinates mounted a mean ELISA titer of 4.6 log_10_ and the titers for JH18 vaccinated mice was 4.7 log_10._ All vaccinated mice demonstrated strong seroconversion, as determined by these significant antibody titers.

### IGF-1 plasma levels

**Figure 5:**
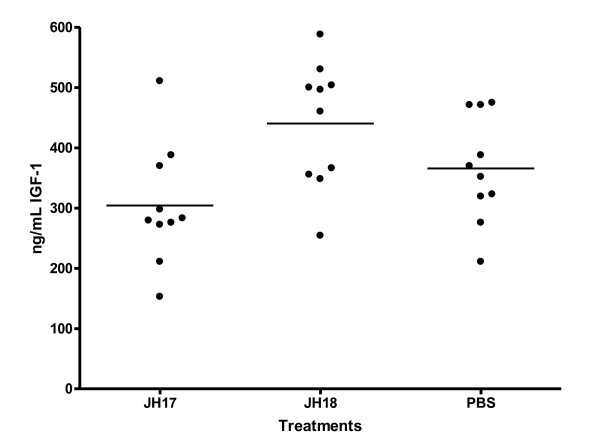
Plasma levels of IGF-1 (ng/mL) of treatment group mice.

IGF-1 levels were determined by a commercial ELISA assay specifically designed for the rat/mouse model. Treatment group mean IGF-1 values were: 304.2 ng/mL (JH17), 440.4 ng/mL (JH18) and 365.6 ng/mL (PBS controls) Figure 5. Statistical analyses either by parametric or non parametric (one-tailed Mann Whitney) methods did not indicate a statistically significant differences between any two treatment groups.

### Insulin plasma levels

Insulin levels were determined by a commercial ELISA assay specifically designed for the mouse model. Treatment group mean insulin values were: 1.552 ng/mL (JH17), 1.508 ng/mL (JH18) and 1.468 ng/mL (PBS controls). Statistical analyses either by parametric or non -parametric (one-tailed Mann Whitney) methods did not indicate a statistically significant differences between any two treatment groups. Based upon these results, vaccination did not inhibit insulin secretion, as compared to normal Insulin levels in PBS controls.

### Effect of vaccination on tested parameters

Data in Table 1 summarizes the parameters of baseline weight, food consumption, chimeric-somatostatin antibodies responses and IGF-1 levels in all mouse treatment groups. Table 2 summarizes the levels of insulin in mouse plasma post- second vaccination.

**Table 1:** Summary results for mouse treatment groups at study end

**Treatments**	**Baseline Body Weight,%**	**Cumulative food Intake**			**Somatostatin ELISA antibody titers (log**_**10**_**)**	**Mean IGF-1, ng/mL**
		**Total g**	**R**^2^	**Slope**		
**JH 17**	107.1***	105.2	0.99	2.987 ± 0.1225	4.7 ***	304.2
**JH 18**	104.0***	96.6	0.99	2.849 ± 0.1301	4.6 ***	440.4
**PBS controls**	115.5	113.8	0.98	3.038 ± 0.2002	2 .0	365.6

*** P < 0.0001 versus PBS controls. R^2^ and slope were determined by linear regression from data in Figure 3.

**Table 2:** Plasma Insulin levels for mouse treatment groups at study end

**Treatments**	**Insulin, ng/mL**	**SEM**	**Passed normality test**
**JH 17**	1.552*	**0.226**	**Yes**
**JH 18**	1.508*	**0.187**	**Yes**
**PBS controls**	**1.468**	**0.281**	**No**

* *P* > 0.05 versus PBS controls.

## Discussion

The use of therapeutic vaccines in medicine is already in practice for treatment of human melanoma and prostate cancer. Following these novel products are other vaccines for treating various pathological conditions and metabolic diseases [15]. A therapy to treat and manage human obesity by vaccination would afford physicians with a common and accepted medical procedure to treat a globally widespread human disease condition. This therapy can be accomplished without drugs or surgical procedures.

Our approach in designing a therapeutic vaccine targeting the counter-regulatory hormone somatostatin is based on the highly antigenic carrier protein chloramphenicol-acetyl transferase (CAT) which has been made inactive by double site mutations [13,16]. Coupled with the enhanced antigenicity of the chimeric-somatostatin, adjuvants have been added which contain metabolizable oils, a polyacrylic polymer and vegetable polysaccharides [14]. These vaccines have been previously tested in normal, non-obese mice for safety and efficacy. The JH17 and JH 18 adjuvants were demonstrated to be safe via intraperitoneal injection in a 0.5 mL dose. The chimeric-somatostatin protein was demonstrated to be highly antigenic by measuring mouse weight gain over a 7 day period and demonstrating a 110% to 130% weight increase compared pre-vaccination weights (data not shown).

The use of somatostatin vaccines for treatment of obesity is based upon previous studies using GH therapy [5,6] and the accepted endocrine model of somatostatin to down-regulate growth hormone releasing hormone (GHRH), GH and IGF-1 [17]. By reversing the effects of somatostatin by immuno- regulation, increased levels of GHRH, GH and IGF-1 were anticipated with increased metabolism.

The DIO mouse model was chosen as it is easily adaptable for vaccination studies and is well characterized for obesity treatments. Since the effective dose in the C57BL/6 J male mouse was not previous determined, we utilized the same dose as we had proven effect in non-obese mice (1 mg protein/mL in a 0.5 mL dose). In this study, this dose volume would represent approximately 1.6% of the mouse body weight. This dose volume would translate to 1.6 liters of vaccine for a 100 kg human, if volume were the determining factor of the vaccine’s effectiveness. In a recently completed pig study, a 1 mL dose containing 0.5 mg of vaccine antigen demonstrated effectiveness in 91 kg pigs as assessed by positive seroconversion and enhanced IGF-1 levels (unpublished data). Therefore, the vaccine’s effectiveness appears to be more related to immunogen content rather than the volume of the injection. This specific antigen dose relationship was previously described by Lunin [18].

Mice were vaccinated and observed for physiological responses to vaccination with the somatostatin vaccines, presented as weight loss and reduction in body weight gain. The amount of food intake was also monitored as it was important to determine if any continued weight loss was due to lack of eating the 60% Kcal diet. Although there was an initial reduction in food consumption during the first 2 days post-1^st^ vaccination, JH17 and JH 18 mice food intake was not statistically significant from the food consumption of PBS control for the duration of the study.

Previous experience with the chimeric-somatostatin vaccines indicated antibody responses are first demonstrable at 4 to 10 days post-vaccination (IgM and IgG subclasses). At these time periods, specific antibody binds to somatostatin and attenuates, but do not completely eliminate the counter-inhibitory effects of the hormone. The main increase of GH and IGF-1 follows this initial antibody production and expected metabolic changes are observed [13]. Since the biological activity of the anti-somatostatin antibodies are short-lived, and are not re-stimulated by endogenous somatostatin, the vaccine effectiveness is similar to repeated drug administration and is not cumulative.

Memory cells responses, as are normally observed with infectious disease vaccines, have not been observed with the chimeric-somatostatin vaccines and each vaccination is likened to a primary dose [18]. Based on these inherent immunological traits of the vaccine, the 2^nd^ vaccination with either JH 17 or JH 18, did not produce a memory response, but rather effected a lesser control on weight gain. In response to the lessened effect, the vaccine-treated mice continued to gain less weight, while consuming the same amount of food as the PBS controls. The second vaccination of 1/5^th^ the amount of protein of the 1^st^ vaccination, can be viewed as a maintenance dose.

Reduction in weight gain can be linked to high anti-somatostatin antibody levels and enhanced IGF-1 quantities in the plasma. Since the samplings of these two markers were conducted at the end of the study, their maximal effectiveness would be anticipated to be during the first week post-vaccination. The demonstration of high levels of anti-somatostatin antibodies at day 39 is indicative of the continued presence of down regulation of somatostatin which is demonstrated by the continued weight gain differentials observed through the study. The antibody titers measured at this one time point, are residual titers and represent the downward part of the antibody effectiveness curve. A similar argument can be made for IGF-1 levels in plasma. It could be postulated the maximal IGF-1 would be seen in the first week following vaccination or revaccination. Even in the presence of these elevated anti-somatostatin antibodies, plasma insulin levels in vaccinated mice were similar to control mice, and demonstrated no statistically significant differences.

The two adjuvants reported in this study were chosen for usefulness in human vaccines. Another adjuvant, approved for use in livestock and containing mineral oil, was also tested in the DIO mouse model (data not shown). The latter adjuvant produced a much more heightened weight loss result accompanied by dehydration, lethargy and death in 8/10 mice treated. Based upon the comparison of these vaccines, it was determined that the chimeric-somatostatin vaccine effects are related to both antigen dose and adjuvant type.

The differences between the JH17 and JH18 adjuvants reside only in their plant polysaccharide content, tragacanthin and arabinogalactan. The results obtained in this study were not statistically significant in any tested parameter between the two vaccine groups. Comparing the two adjuvants, the observation of JH18 producing percent of baseline body weight and enhanced IGF-1 are indicative but not predictive of a superior adjuvant effect of the two polysaccharide additions. In future studies, only a single adjuvant will be utilized and sham vaccinated controls will receive the same adjuvant, but without the chimeric protein included. In this design, the observable food reduction for 2 days after the 1^st^ vaccination can be determined to be a property of the adjuvant or the total vaccine compound.

In summary, DIO mice treated with 2 vaccine formulations, containing the same dose of chimeric-somatostatin protein, were effective in reducing weight gain and reducing final body weight percentage versus baseline weights, when compared to PBS control mice. The vaccine effect was observable even while the mice were continuously fed a 60% Kcal fat diet. The vaccination effects did not significantly reduce cumulative food consumption and was confirmed by residual anti-somatostatin antibodies in mouse plasma at the study’s end. Measured levels of Insulin in vaccinates were similar to controls, further adding to the vaccine’s safety profile. The final result of the study is the demonstration of the usefulness of treating obesity with vaccination and warrants additional studies and parameter monitoring in other animal models (normal pigs, obese minipigs and obese dogs).

## Competing interests

The author declares no conflict of interest.

## References

[B1] Ogden CL Carroll MD McDowell MA Flegal KM Obesity among adults in the United States— no change since 2003–2004 2007 National Center for Health Statistics, Hyattsville, MD NCHS data brief no 1

[B2] Flegal KM Graubard BI Williamson DF Gail MH Excess deaths associated with underweight, overweight, and obesity JAMA 2005 293 1861 1867 10.1001/jama.293.15.1861 15840860

[B3] Daughaday WH Hall K Raben MS Salmon WD Brande JL Wyk JJ Somatomedin: proposed designation for sulphation factor Nature 1972 235 107 109 10.1038/235107a0 4550398

[B4] Johansen T Malmlof K Treatment of obesity using GH Metab Syndr Relat Dis 2006 4 57 69 10.1089/met.2006.4.57 18370772

[B5] Franco C Bengtsson BA Johannsson G The GH/IGF-1 axis in obesity: physiological and pathological aspects Metab Syndr Relat Dis 2006 4 51 56 10.1089/met.2006.4.51 18370771

[B6] Vickers MH Ikenasio BA Breier BH Adult growth hormone treatment reduces hypertension and obesity induced by an adverse prenatal environment J Endocrinol 2002 175 615 623 10.1677/joe.0.1750615 12475373

[B7] Patel YC Srikant CB Somatostatin and its receptors Adv Mol Cell Endocrinol 1999 3 43 73

[B8] Spencer GS Hormonal systems regulating growth. A review Livest Prod Sci 1985 12 31 46 10.1016/0301-6226(85)90038-7

[B9] Estrada A Laarveld B Bing L Redmond M Induction of systemic and mucosal immune responses following immunization with somatostatin-avidin complexes incorporated into Iscoms Immunol Invest 1995 24 819 828 10.3109/08820139509060709 8543345

[B10] Fuller MF The Encyclopedia of Farm Animal Nutrition 2004 CABI Publishing 280 285

[B11] Clee SM Attie AD The genetic landscape of type 2 diabetes in mice Endocr Review 2006 2 48 83 10.1210/er.2006-0035 17018838

[B12] The Jackson Laboratory Type 2 diabetes and obesity research and the laboratory mouse 2007 A Jackson Laboratory Resource Manual

[B13] Mendelsohn AR Haffer KN Larrick J Chloramphenicol acetyl transferase (CAT)-defective somatostatin fusion protein and uses thereof 2010 United States Patent, US 7,722,881B2

[B14] Haffer KN Larrick J Mendelsohn AR Compositions and methods for enhanced somatostatin immunogenicity in the treatment of growth hormone and insulin-like growth factor one deficiency 2011 United States Patent Application Publication, US 2011/0195080A1

[B15] Shirvill J Innovations and Opportunities in Therapeutic Vaccines: Technology platforms, key players, and early pipeline candidates 2010 Business Insights

[B16] Lewendon A Murray IA Shaw WV Gibbs MR Leslie AG Replacement of catalytic histidine-195 of chloramphenicol acetyl transferase. Evidence for a general role for glutamate Biochemistry 1994 33 1944 1950 10.1021/bi00173a043 7906544

[B17] Guillemin R Gerich JE Somatostatin: Physiological and clinical significance Ann Rev Med 1976 27 379 388 10.1146/annurev.me.27.020176.002115 779605

[B18] Lunin VG Sergienko OV Khodun ML Bader LB Karpov VA Tikhomento TI Chimeric somatostatin containing protein encoding DNA, plasmids of expression, methods for preparing chimeric protein, strain-producers, immunogenic composition, method for increasing the productivity of farm animals 2001 United States Patent, US 6,316,004B1

